# Molecular Detection of *Pentastiridius leporinus*, the Main Vector of the Syndrome ‘Basses Richesses’ in Sugar Beet

**DOI:** 10.3390/insects13110992

**Published:** 2022-10-28

**Authors:** René Pfitzer, Mark Varrelmann, Georgia Hesse, Omid Eini

**Affiliations:** 1 Institute of Sugar Beet Research, Holtenser Landstraße 77, 37079 Göttingen, Germany; 2 Agricultural Entomology, Department of Crop Sciences, Faculty of Agricultural Sciences, University of Göttingen, Grisebachstrasse 6, 37077 Göttingen, Germany

**Keywords:** Cixiidae, mitochondrial cytochrome oxidase I, phylogeny, simple DNA preparation, species-specific primers

## Abstract

**Simple Summary:**

*Pentastiridius leporinus* is the main vector of a new and fast spreading disease, the syndrome ‘basses richesses’ (SBR) in sugar beet. SBR causes high sugar content and yield losses in Central Europe. Monitoring of this insect vector based on morphological identification is challenging as two other cixiid species *Reptalus quinquecostatus* and *Hyalesthes obsoletus* with similar external characters are known to additionally appear in sugar beet fields. In this study, a PCR-based method is provided for simple and reliable detection of *P. leporinus* collected via sweep nets and sticky traps. This method also detects eggs and all nymphal stages and differentiates this vector from the most common Auchenorrhyncha species occurring in sugar beet fields. Furthermore, the phylogenetic relationship of these morphologically close cixiid species was investigated based on the mitochondrial cytochrome oxidase I gene (*COI*).

**Abstract:**

Monitoring of *Pentastiridius leporinus* (Hemiptera: Auchenorrhyncha: Cixiidae), representing the main vector of the syndrome ‘basses richesses’ (SBR) disease in sugar beet is based on morphological identification. However, two other cixiid species, *Reptalus quinquecostatus* and *Hyalesthes obsoletus* with similar external characters are known to appear in sugar beet fields and are challenging to be distinguished from *P. leporinus*. We present a PCR-based method for species-specific detection of both male and female *P. leporinus*, directly after sweep net collection or after up to 18 months long term storage on sticky traps. Two methods of DNA template preparation, based on a commercial extraction kit or on simple grinding in phosphate-buffered saline (PBS) were compared. The latter method was also established for eggs and all five nymphal instars of *P. leporinus* from a rearing. Furthermore, in silico primer analysis showed that all Auchenorrhyncha species including far related species reported from sugar beet fields can be differentiated from *P. leporinus*. This was PCR-confirmed for the most common Auchenorrhyncha species from different German sugar beet fields. Sequence analysis of the *P. leporinus* mitochondrial cytochrome oxidase I gene (*COI*) amplicon showed a close relationship to *COI* from *P. beieri* but separated from the *Reptalus* and *Hyalesthes* species which are grouped into the same family Cixiidae. We present a sensitive, cost- and time-saving PCR-based method for reliable and specific detection of eggs and all nymphal instars, as well as male and female *P. leporinus*, after different methods of planthopper collection and template DNA template preparation that can be used in large scale monitoring assays.

## 1. Introduction

The syndrome ‘basses richesses’ (SBR) is a fast-spreading sugar beet (*Beta vulgaris*) disease leading to up to 5% absolute sugar content loss and severe yield reduction of the taproot [1,2,3]. Since the first report in 1991, a fast spread of SBR occurred in eastern France, and 1800 ha were infected in 2004 [4]. The first detection of SBR in German sugar beet fields was in 2009, whereas the estimated area of infestation was more than 16,000 ha in 2018 [5]. A further spread of the disease into additional sugar beet growing regions of Germany was reported by Behrmann et al. [6]. Additionally, SBR appeared in sugar beet fields (5000 ha) in Switzerland in 2021 [7]. Two SBR causal agents were described, the γ3-proteobacterium ‘*Candidatus* Arsenophonus phytopathogenicus’ (here called: ‘SBR proteobacterium’) and the stolbur phytoplasma (16SrXII group) ‘*Candidatus* Phytoplasma solani’ [2,4,8,9,10]. Both pathogens are exclusively transmitted by planthoppers [1,3].

The most important SBR vector in sugar beet fields is *Pentastiridius leporinus* (Hemiptera: Auchenorrhyncha: Cixiidae), due to its high population densities, infection rates and the ability to transmit both pathogens to sugar beet plants [1,3,5,10,11]. Additionally, female adult *P. leporinus* can vertically transmit the SBR proteobacterium to their offspring [12]. This allows maintenance of the pathogen over various generations in a rearing under lab conditions [13]. The only known natural host plant of *P. leporinus* is reed (*Phragmites australis*) [14]. Recently, *P. leporinus* host-shifted to sugar beet and winter wheat (*Triticum aestivum*) or barley (*Hordeum vulgare*) crop rotations. This phenomenon together with increasing *P. leporinus* populations led to a significant SBR spread [8,15,16].

Several Auchenorrhyncha species from various taxonomic families and subfamilies have been collected in sugar beet fields [5,11,17]. Among them, there are two closely related cixiid planthoppers. Sémétey et al. [11] reported, that adult *Reptalus* sp. were present in sugar beet fields in the French regions, Burgundy, and Franche-Comté. In German field studies in Baden-Württemberg in 2018, *R. quinquecostatus* was the most common cixiid planthopper species after *P. leporinus* [5,17]. No other species besides *R. quinquecostatus* were found in sugar beet within the genus *Reptalus*. These observations were confirmed by sampling from different sugar beet fields in Baden-Württemberg in 2019 and 2020 [17,18].

**Figure 1:**
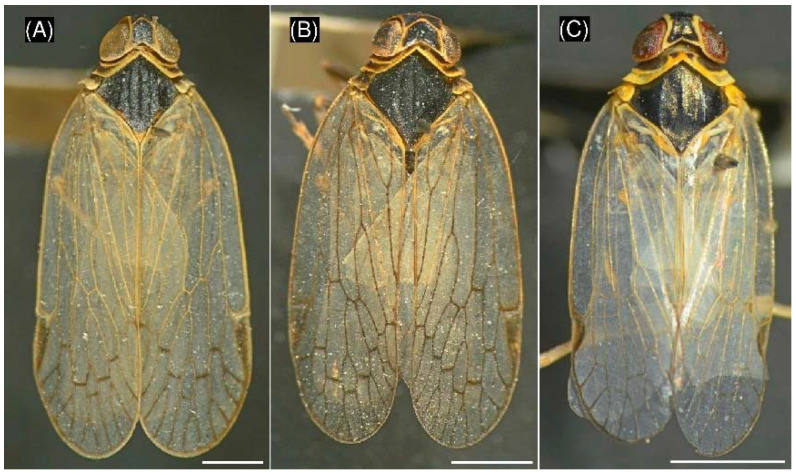
Morphology of three close cixiid planthoppers showing hyaline or transparent wings, the fore wings are characterised by a roof-shaped resting position, and the mesonotum has five keels. (**A**) *Pentastiridius leporinus*, (**B**) *Reptalus quinquecostatus*, (**C**) *Hyalesthes obsoletus*. Scale bar represents 1 mm.

The second species, *Hyalesthes obsoletus* (Hemiptera: Cixiidae) is an important vector for several plant diseases e.g., “bois noir” [19,20,21], potato stolbur disease [22], lavender decline [23] and the maize redness disease [24] by transmission of *Ca*. P. solani. Sémétey et al. [11] and Bressan et al. [1] reported this planthopper species from French sugar beet fields and demonstrated that *H. obsoletus* is a potential vector of the SBR disease under controlled environmental conditions. *H. obsoletus* was collected from different sugar beet fields in Germany (Baden-Württemberg) between 2018 and 2020 [5,17,18]. *R. quinquecostatus* and *H. obsoletus* can be hardly distinguished from *P. leporinus* by morphological traits (Figure 1). These three species have hyaline or transparent wings, the fore wings are characterised by a roof-shaped resting position, the mesonotum has five keels and the absence of a post-tibial calcar at the hind legs [14]. These species are also closely related in a phylogenetic analysis based on morphological traits [25]. Due to the fast spread of the SBR disease, *P. leporinus* monitoring is much needed. Usually, adult Auchenorrhyncha collection is carried out with sweep netting or sticky traps [26]. *P. leporinus* eggs or nymphs can be directly collected from soil [12]. Sticky traps represent an important tool to monitor the vector spread, but glue removal and species classification of planthoppers by morphological traits are time-consuming and error prone [27,28]. Further problems of traditional species identification are the need for highly skilled and experienced personnel [28,29]. This clearly limits the throughput in practical monitoring [28]. More, morphological keys for *P. leporinus* identification at the species level are exclusively described for male adults [14,30]. To our knowledge, a morphological method is lacking to discriminate female adults or immature *P. leporinus* including eggs from other cixiid species. Molecular methods can be used to support or substitute morphological species identification [27,28,31,32]. Hebert et al. [33] established the use of DNA barcoding based on mitochondrial cytochrome oxidase I gene (*COI*) sequences for taxonomic insect identification. The *COI* gene was used for identification of planthoppers in the genera *Reptalus* or *Hyalesthes* using species-specific primers [27,29], for sequence analysis of several cixiid species including *P. leporinus*, *R. quinquecostatus*, and *H. obsoletus* [16,27,29] or for phylogenetic analysis of cixiid and delphacid planthoppers including *P. leporinus*, *Reptalus cuspidatus*, and *H. obsoletus* [34].

The aim of this study was to establish a species-specific, inexpensive and time-saving PCR detection for *P. leporinus* eggs, immature stages and both male and female adults allowing differentiation from two other closely related species (*R. quinquecostatus* and *H. obsoletus*). In addition, sequence analysis showed that the designed primers enable differentiation of *P. leporinus* from all other Auchenorrhyncha species that have been described from sugar beet fields, including morphologically and taxonomically close as well as distantly related species. Furthermore, two common sources of insect collections (sweep netting with direct preservation or sticky trap collection) and two methods of template DNA preparation were evaluated. The evolutionary relationships based on the *P. leporinus* partial sequence of the *COI* gene confirmed the relationship between closely and distantly related Auchenorrhyncha species.

## 2. Materials and Methods

### 2.1. Planthopper Collection and Morphological Identification

Closely related cixiids (adult *P. leporinus*, *R. quinquecostatus*, and *H. obsoletus*) were field-collected with sweep nets or yellow sticky traps during summer 2020 from several locations in Germany (Baden-Württemberg, Rhineland-Palatinate, and Saxony). Morphological identification of the sweep net collected insects was carried out within 24 h after collection. Sticky traps 10 cm × 25 cm (‘Gelbe Insekten-Leimtafeln’, Aeroxon Insect Control GmbH, Waiblingen, Germany) were collected after seven days and transferred into polypropylene cards (‘office discount Sichthüllen DIN A4 glasklar glatt 0,12 mm’, office discount GmbH, Neufahrn bei München, Germany). Sticky trap collected specimens were stored on the traps for 14–18 months (long term) at room temperature (15–25 °C) before morphological identification was carried out.

The most common Auchenorrhyncha species reported from German sugar beet fields (species are provided in Section 2.4) were collected during summer 2020 and stored on sticky traps for 1–2 weeks (short term) before morphological identification was performed [17].

Morphological identification of planthoppers was carried out with a stereomicroscope according to the taxonomic key of Biedermann & Niedringhaus [30]. Family and genus of individual female adult specimens were identified by observation of wings, pronotum, mesonotum, postnotum, and tarsus. Furthermore, the genital structures of male adults were evaluated to allow morphological identification at the species level. Hereafter, sweep net collected specimens were preserved in 96% ethanol and at −20 °C and sticky trap collected specimens with glue attached were preserved in 60% or 70% ethanol at room temperature until further use. Additionally, *P. leporinus* eggs and all five nymphal instars were obtained from a rearing on sugar beet [13]. Developmental stages of nymphs were determined under a stereomicroscope according to the key of Pfitzer et al. [13], before specimens were preserved in 96% ethanol at −20 °C until further use.

### 2.2. Template DNA Preparation

Detailed information about experimental samples is provided in Supplementary Table S1. Insect DNA templates were obtained either by using ‘DNeasy Blood & Tissue Kit’ (QIAGEN GmbH, Hilden, Germany) according to the manufacturer’s instructions or simply by crushing the insects in phosphate-buffered saline (PBS) as described by Priti et al. [35] with slight modifications. Individual insects were transferred into 1.5 mL microcentrifuge tubes with 60 μL (*Stictocephala bisonia* adults: 120 μL) or 30 μL (eggs and nymphs) PBS (pH 7.4), then crushed with a sterile micropestle and incubated at 100 °C for 10 min. Additionally, the tubes were centrifuged for 10 min with 13,500× *g* at room temperature. The supernatant (template DNA concentrations are provided in Section 2.3) was used as a PCR template. DNA quality and quantity were analyzed with a spectrophotometer (‘DeNovix DS-11′, DeNovix Inc., Wilmington, DE, USA). To avoid DNA contamination between samples, we used a single undamaged insect for DNA preparation. Furthermore, to avoid DNA degradation, DNA extracts by means of DNeasy Blood & Tissue Kit were diluted in AE buffer and PBS extracted DNA were used in a short time, within a week.

### 2.3. Primer Design and PCR Conditions

*COI* sequences of *P. leporinus, R. quinquecostatus,* and *H. obsoletus* were obtained from the NCBI database (National Center for Biotechnology Information, U.S. National Library of Medicine, Rockville Pike, MD, USA) and multiple-aligned with the software BioEdit 7.2 [36] for primer design. *COI* sequences of *P. leporinus* were also compared to each two additional representative taxonomically close *Reptalus* and *Hyalesthes* species (*R. melanochaetus, R. panzeri, H. luteipes,* and *H. scotti*) for species-specific primer design. The specific *P. leporinus* fw1 and rv1 primers were designed to have no miss-match with the *COI* gene of *P. leporinus* but show miss-match with the *COI* gene of the closely related species.

Furthermore, the specificity of the designed primers was tested in silico on all Auchenorrhyncha species reported to occur in sugar beet fields [5,11,17] for which *COI* sequences were available at the NCBI database. A list of primers (Table 1) is provided.

Species-specific PCR reactions with *P. leporinus* fw1 and rv1 primers were carried out in a final volume of 20 μL, consisting of 10 μL ‘DreamTaq PCR Master Mix (2X)’ (Thermo Fisher Scientific, Waltham, MA, USA), 0.5 μM of each primer and 13–27 ng (eggs), 80–270 ng (nymphs), 25–150 or 2–8 ng (sweep net or sticky trap collected adults after kit extraction), and 7–73 ng (adults after preparation in PBS) template DNA. PCR conditions were 98 °C for 2 min, 30 cycles at 95 °C for 30 s, 56 °C for 25 s and 72 °C for 25 s and a final step at 72 °C for 10 min.

A ~1000 bp fragment of the *COI* gene was amplified with primers Ron and Calvin [37] and used as a control for DNA quality. Another PCR protocol was used for amplification of a ~1000 bp fragment of the *COI* region from *R. quinquecostatus* and *H. obsoletus* with primers UEA3 and UEA8 according to Lunt et al. [38]. PCR reactions were carried out in a mixture with a final volume of 20 μL, consisting of 10 μL DreamTaq PCR Master Mix (2X), 0.5 μM of each primer and the same (UEA3 and UEA8) or double (Ron and Calvin) template DNA concentrations compared to species-specific PCR (described above). Thermocycling conditions consisted of 95 °C for 2 min, 35 cycles at 95 °C for 30 s, 51 °C (Ron and Calvin) or 54 °C (UEA3 and UEA8) for 30 s and 72 °C for 75 s and a final 72 °C step for 10 min.

PCR products were separated on 1 % agarose gels and stained with ‘Gelred’ (Biotium, Landing Pkwy, CA, USA) next to a ‘GeneRuler 1 kb DNA ladder’ (Thermo Fisher Scientific, Waltham, MA, USA). PCR products were sequenced (Microsynth Seqlab GmbH, Göttingen, Germany) and the data were used in phylogenetic analysis. Furthermore, *COI* sequences were aligned to sequences from the NCBI database to support morphological determination (see Section 2.1).

### 2.4. Application to Adult and Immature Specimens

The specificity of *P. leporinus* fw1 and rv1 primers was tested on both male and female adults of *P. leporinus*, *R. quinquecostatus*, and *H. obsoletus* using the two template preparation methods. Furthermore, these primers were also tested for detection of eggs and all nymphal instars of *P. leporinus* after PBS template preparation. For these assays, we had no access to *R. quinquecostatus* and *H. obsoletus* immature specimens, so only adults were used as the negative control.

Additionally, PCR specificity tested for the most common Auchenorrhyncha species reported from German sugar beet fields [17] including morphologically and taxonomically close and distant species from various families (Cixiidae, Delphacidae, Membracidae, and Cicadellidae). These species included: *P. leporinus*, *Empoasca pteridis*, *Empoasca affinis*, *Cicadula placida*, *Orientus ishidae*, *R. quinquecostatus* (closely related), *Psammotettix alienus*, *Empoasca decipiens*, *Fieberiella florii*, *Javesella pellucida*, *S. bisonia*, and *Javesella obscurella*. *J. obscurella* (family: Delphacidae, 20th most common Auchenorrhyncha species from sugar beet) was added, due to absence of *COI* mismatches on the 3′ end with *P. leporinus* fw1 primer (see below).

### 2.5. Evolutionary Relationships

The amplified part of the *COI* (341 bp in size) of *P. leporinus* was sequenced and applied for BLAST search. Ten representative entries from *Pentastiridius* spp., *Reptalus* spp., and *Hyalesthes* spp. were selected to test their phylogenetic relationship using the neighbor-joining method [39]. *Catonia carolina* (family: Achilidae) and *Tettigometra virescens* (family: Tettigometridae) were used as outgroups. The percentage of replicate trees in which the associated taxa clustered together in the bootstrap test (1000 replicates) are shown next to the branches [40]. Furthermore, evolutionary divergence between sequences was estimated and the number of base substitutions per site from between the sequences is shown. The evolutionary distances were computed using the Maximum composite likelihood method [41] and are in the units of the number of base substitutions per site. All ambiguous positions were removed for each sequence pair (pairwise deletion option). Evolutionary analysis was conducted using MEGA X [42]. Similarly, the part of the *COI* gene (ca. 1000 bp depending on the species) amplified from *P. leporinus*, *R. quinquecostatus*, and *H. obsoletus* using universal primers, was sequenced, and used in a BLAST search. Each one representative *COI* sequence from the NCBI database per Auchenorrhyncha family and subfamily reported from sugar beet fields [17] was aligned and used for phylogenetic analysis, including another taxonomically close family Delphacidae [43]. This was to show whether the amplified *COI* sequence is helpful to group these closely and far related species.

## 3. Results

### 3.1. Species-Specific Primer Design

**Figure 2:**
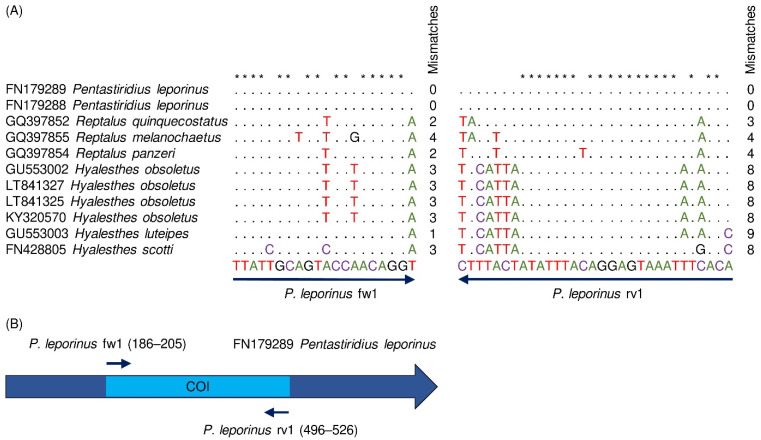
Alignment of the specific primers (*P. leporinus* fw1 and rv1) to the *COI* gene of *Pentastiridius leporinus* and different members of *Reptalus* spp. and *Hyalesthes* spp. (**A**) Dots mark identical nucleotides in the specific primers and the analyzed sequences. Asterisks mark the positions of conserved nucleotides within primer sequences. Nucleotide mismatches between primers and analyzed sequences are highlighted with letters and numbers indicated for each sequence. (**B**) The schematic map represents the location of specific primers on the partial *COI* gene. Arrows represent the locations of the primers which amplify a 341 bp fragment (light blue) on *P. leporinus COI* sequence available from the NCBI database (dark blue).

in silico analysis was conducted to test the specificity of the newly designed *P. leporinus* fw1 and rv1 primers towards the *COI* gene of various species within the genera *Pentastiridius*, *Reptalus*, and *Hyalesthes* available from the NCBI database. No mismatches to the primers were observed for the different *P. leporinus* sequences (FN179289, FN179288, Figure 2A). However, one to four mismatches to the forward and three to nine mismatches to the reverse primer were observed in the sequences of each three *Reptalus* and *Hyalesthes* species, respectively. Each sequence displayed at least one mismatch at the 3′ ends of both primers and the mismatches accumulated at the 3′ ends (Figure 2A). The primer positions on *P. leporinus COI* are displayed in Figure 2B. in silico a 341 bp PCR product was obtained.

Alignment of the specific primers to the *COI* gene of various Auchenorrhyncha genera or species, reported from sugar beet fields, showed 2 to 14 mismatches to the fw1 primer and 3 to 23 mismatches to the rv1 primer (Supplementary Figure S1). Most of the mismatches occurred at the primers 3′ end. Two exceptions (*J. obscurella* and *N. campestris*), where the mismatches to *P. leporinus* fw1 primer were not located at the 3′ ends, were observed. However, seven and ten mismatches, respectively, were observed for these two species to *P. leporinus* rv1 primer and at least two of the mismatches were located at the 3′ ends. Therefore, distantly related Auchenorrhyncha species from sugar beet fields may not be detected with these specific primers.

**Figure 3:**
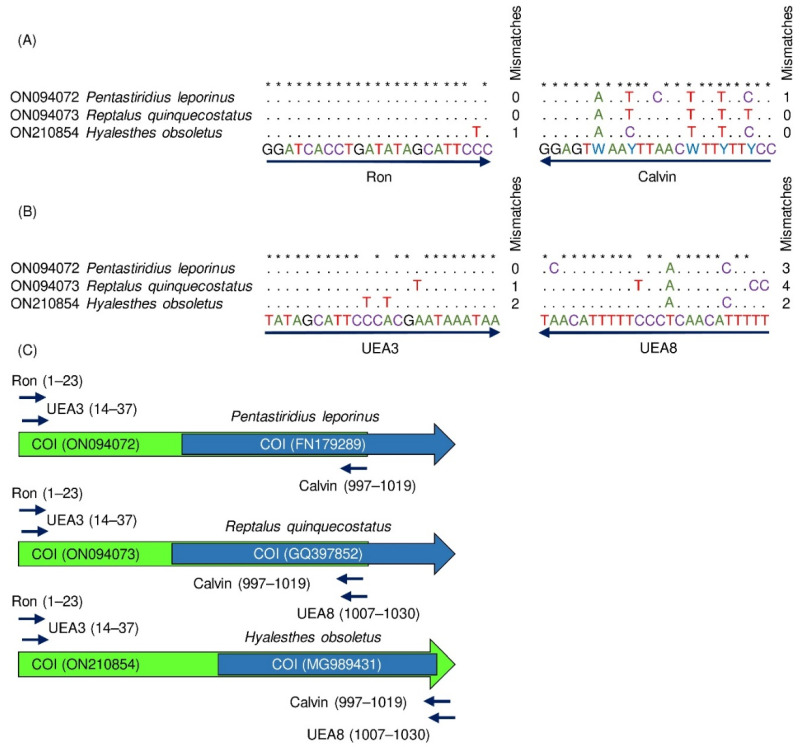
Alignment of the universal primers (Ron/Calvin and UEA3/UEA8) to *P. leporinus*, *R. quinquecostatus*, and *H. obsoletus COI* sequences and primer location within the *COI* gene. Alignment of (**A**) Ron/Calvin and (**B**) UEA3/UEA8 primers to the *COI* gene of *P. leporinus*, *R. quinquecostatus*, and *H. obsoletus*. Dots mark identical nucleotides in the primers and the analyzed sequences. Asterisks mark the positions of conserved nucleotides within primer sequences. Nucleotide mismatches between primers and analyzed sequences including numbers are indicated for each sequence. (**C**) *P. leporinus*, *R. quinquecostatus*, and *H. obsoletus COI* schematic maps. Arrows represent the locations of the primers on the *COI* gene and show that Ron and UEA3 as well as Calvin and UEA8 partly overlapped. The green color shows the fragment that was amplified and sequenced in this study for each species. The blue color shows the available sequence from the NCBI database.

Furthermore, the universal *COI* primer pairs Ron/Calvin and UEA3/UEA8 were aligned to the *P. leporinus*, *R. quinquecostatus*, and *H. obsoletus COI* sequences. Ron and Calvin primers were used for molecular detection of cixiids according to Urban et al. [44] and UEA3 and UEA8 primers were designed for general *COI* amplification of hemipteran insects [38]. The numbers and positions of mismatches are shown in Figure 3A,B. Primer positions on the *COI* sequences are represented in Figure 3C. Ron and Calvin primers each had a maximum of one mismatch with *P. leporinus*, *R. quinquecostatus*, and *H. obsoletus COI*. UEA8 primer had three mismatches with *P. leporinus COI* (one mismatch on the next-to-last nucleotide at the 3′ end, Figure 3B) which is expected to interfere with PCR amplification (Figure 3C).

### 3.2. PCR Validation on Adult Planthoppers

**Figure 4:**
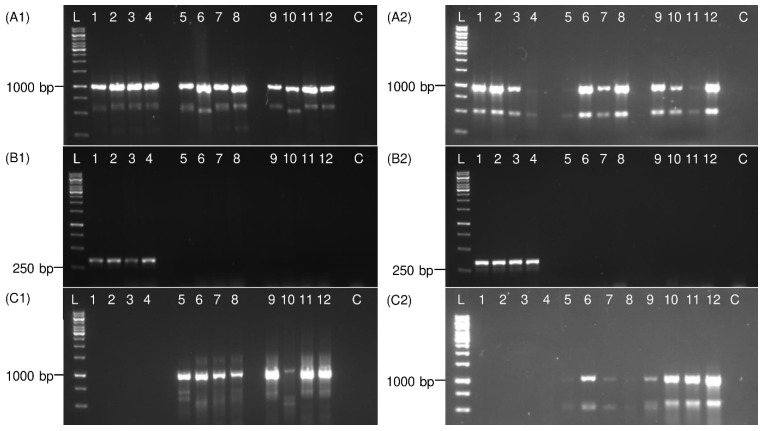
Electrophoretic patterns of PCR products show specific *P. leporinus* detection. Total DNA was extracted from adult specimens collected from either sweep nets (**A1**,**B1**,**C1**) or sticky traps (**A2**,**B2**,**C2**) using a blood and tissue kit. In panels (**A1**,**A2**), universal Ron and Calvin primers, in panels (**B1**,**B2**), specific *P. leporinus* primers and in panels (**C1**,**C2**), universal UEA3 and UEA8 primers were used for PCR. Lanes 1, 2 represent *P. leporinus* male adult samples; 3, 4 *P. leporinus* female adult; 5, 6 *R. quinquecostatus* male adult; 7, 8 *R. quinquecostatus* female adult; 9, 10 *H. obsoletus* male adult; 11, 12 *H. obsoletus* female adult; C: Negative control (water). The sizes of amplicons are shown on the left side and compared with 1 kb ladder (L).

The specificity of *P. leporinus* fw1 and rv1 primers was tested on DNA templates, prepared with a DNeasy Blood & Tissue Kit, from male and female adult *P. leporinus*, *R. quinquecostatus*, and *H. obsoletus*. In the specific *P. leporinus* PCR, 100% of the *P. leporinus* specimens and no unspecific sample were detected (Figure 4). However, in the general PCR using universal primers (Ron and Calvin), for both sweep net and sticky trap collected specimens, all samples were detected. Notably, 25% of sticky trap collected insects produced only weak bands. Furthermore, in the general *COI* PCR using UEA3 and UEA8 primers, no DNA amplification was observed for *P. leporinus* specimens but 100% of the *R. quinquecostatus* and *H. obsoletus* specimens produced amplicons. However, 50% of the PCR products obtained from sticky trap collected insects were rather weak (Supplementary Figure S2).

**Figure 5:**
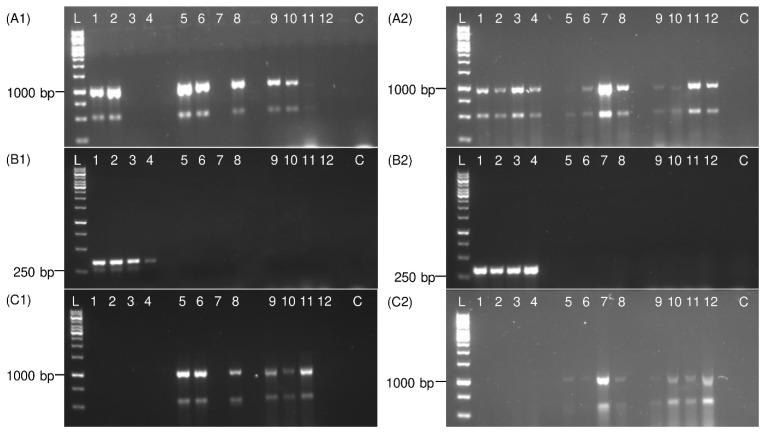
Electrophoretic patterns of PCR products show specific *P. leporinus* detection from PBS extracts. Adult specimens were collected from either sweep nets (**A1**,**B1**,**C1**) or sticky traps (**A2**,**B2**,**C2**). In panels (**A1**,**A2**), universal Ron and Calvin primers, in panels (**B1**,**B2**), specific *P. leporinus* primers and in panels (**C1**,**C2**), universal UEA3 and UEA8 primers were used for PCR assay. Lanes 1, 2 represent *P. leporinus* male adult samples; 3, 4 *P. leporinus* female adult; 5, 6 *R. quinquecostatus* male adult; 7, 8 *R. quinquecostatus* female adult; 9, 10 *H. obsoletus* male adult; 11, 12 *H. obsoletus* female adult; C: Negative control (water). The sizes of amplicons are shown on the left side and compared with 1 kb ladder (L).

Amplification of *COI* fragments from PBS extracts is shown in Figure 5. PBS extracts had a lower quality, compared with DNeasy Blood & Tissue Kit DNA extracts (data not shown). A part of the *COI* was amplified from 75% of the sweep net and 100% of the sticky trap collected specimens in the general *COI* PCR with Ron and Calvin primers, however 25% of the sticky trap collected samples produced weak bands. In specific *P. leporinus* PCR, 100% of *P. leporinus* specimens and none of the other samples were detected. In the general *COI* PCR with UEA3 and UEA8 primers, DNA from none of *P. leporinus* and 75% (sweep net collected) or 100% (sticky trap collected) of *R. quinquecostatus* and *H. obsoletus* samples were amplified. However, most of the sticky trap collected samples produced rather weak bands. The obtained *COI* sequences in this study from *P. leporinus*, *R. quinquecostatus*, and *H. obsoletus* using universal primers were aligned and the consensus sequences were submitted to the NCBI database (accession numbers ON094072, ON094073, and ON210854).

### 3.3. Detection of Immature Life Stages of P. leporinus

**Figure 6:**
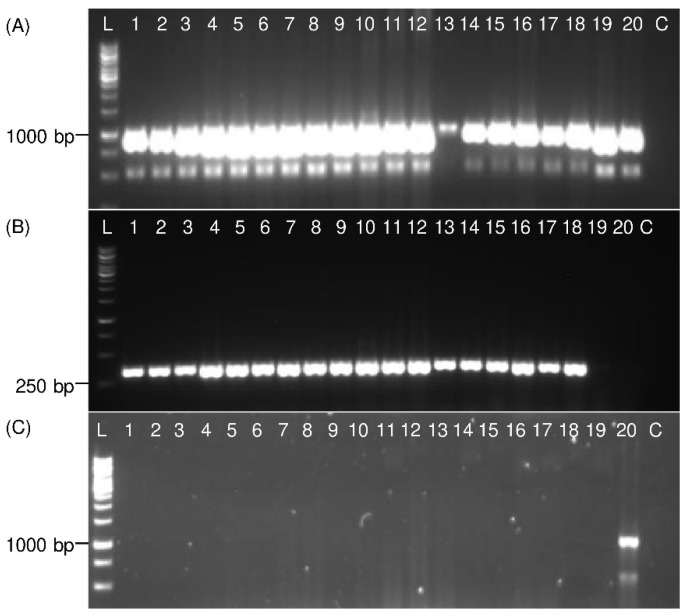
Electrophoretic patterns of PCR products show detection of *P. leporinus* eggs and nymphs from PBS extracts. In panel (**A**), universal Ron and Calvin primers, in panel (**B**), specific *P. leporinus* primers and in panel (**C**), universal UEA3 and UEA8 primers were used for PCR. Lanes 1–3 represent single egg samples; 4–6 first instar; 7–9 second instar; 10–12 third instar; 13–15 fourth instar; 16–18 fifth instar; 19 *R. quinquecostatus* female adult (control); 20 *H. obsoletus* female adult (control); C: Negative control (water). The sizes of amplicons are shown on the left side and compared with 1 kb ladder (L).

The *COI* was amplified from all immature *P. leporinus* specimens, including eggs and all five nymphal stages, using the universal Ron and Calvin primers and specific *P. leporinus* primers (Figure 6). No DNA was amplified from immature specimens using UEA3 and UEA8 primers. In general, single, and clear bands with the expected product size were obtained for all specimens with specific primers.

### 3.4. Detection of Distantly Related Species from Sugar Beet Fields

The specificity of *P. leporinus* primers was tested on the most common Auchenorrhyncha species from German sugar beet fields including closely and distantly related species. No DNA was amplified from other species besides *P. leporinus* with specific *P. leporinus* PCR (Supplementary Figures S2 and S3). In general *COI* PCR with Ron and Calvin primers, a part of the *COI* gene was amplified from *P. leporinus*, *R. quinquecostatus*, *H. obsoletus*, *F. florii*, *J. pellucida*, and *J. obscurella* specimens. The obtained *COI* sequences in this study of *F. florii* and *Javesella* sp. were aligned, and the consensus sequences were submitted to the NCBI database with the accession numbers OP090544, OP068197, and OP103664. In the general *COI* PCR with UEA3 and UEA8 primers, DNA from *R. quinquecostatus*, *H. obsoletus* and one *P. alienus* specimen was amplified.

### 3.5. Evolutionary Relationships

**Figure 7:**
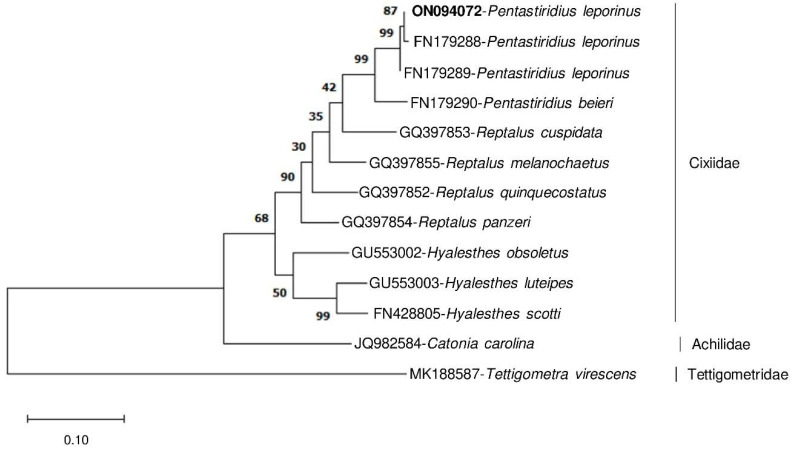
Evolutionary relationships of selected members of Cixiidae based on the partial *COI* sequence amplified from *P. leporinus* using specific primers in this study and *COI* sequences from the NCBI database of closely related species from genus *Pentastiridius* and each three species from two taxonomically close genera *Reptalus* and *Hyalesthes.* The sequence obtained in this study is shown in bold. The percentage of replicate trees in which the associated taxa clustered together in the bootstrap test (1000 replicates) are shown next to the branches. The specifically amplified *COI* fragment is differentiating the closely related species. *Catonia carolina* from the Achilidae family and *Tettigometra virescens* from the Tettigometridae family were used as outgroups.

The phylogenetic relationship of morphologically closely related planthoppers was analyzed based on partial *P. leporinus COI* sequence amplified with specific primers and NCBI *COI* sequences of various species from the genera *Pentastiridius*, *Reptalus*, and *Hyalesthes* (Figure 7). The aim was to test whether the specifically amplified *COI* fragment is sufficient to differentiate those closely related species. Members of the three species clearly separated to different main branches of the phylogenetic tree, confirming morphological differences. Based on this analysis, two *P. leporinus* specimens from Russia (FN179288) and France (FN179289) were phylogenetically closest to the German collections and *P. beieri* was the closest species to *P. leporinus* in this study. Thus, intraspecific genetic distance to *P. leporinus* from Russia (0.0) and France (0.6) was lower than interspecific distance to *P. beieri* (5.1) (Supplementary Table S2). Therefore, the specifically amplified *COI* fragment was variable enough to differentiate *Pentastiridius* spp., *Reptalus* spp., and *Hyalesthes* spp. from each other.

The phylogenetic relationship of closely and distantly related Auchenorrhyncha species reported from sugar beet fields based on the *COI* sequence amplified with universal primer pairs showed that *P. leporinus*, *R. quinquecostatus*, and *H. obsoletus* are closely related and grouped into Cixiidae (Supplementary Figure S4). This confirms the close morphological features for these species. Additionally, these *COI* sequences were useful to clearly differentiate Cixiidae members from Delphacidae and all other representatives from different Auchenorrhyncha families and subfamilies reported in sugar beet fields.

## 4. Discussion

DNA barcoding is a well-established method for insect species identification [31]. It is based on the *COI* sequence comparison with database sequences [31,32]. In addition, insect sequences from internal transcribed spacers (ITS) or *5*.*8S*-*ITS2* rDNA are used for species-specific detection [27,28,45]. Species-specific molecular detection methods are rapid and cost-saving compared to analysis of morphological traits and reduce the risk of misidentification [31,46]. In the presented study, species-specific primers were designed on highly conserved parts of the *COI* gene of the target species as *the COI* gene was variable enough to distinguish *P. leporinus* from all other Auchenorrhyncha species reported from sugar beet fields. Supporting our approach, several studies demonstrated that the *COI* gene was exclusively and successfully used for species-specific insect detection. For example, the *COI* gene was used for species-specific detection of *Reptalus* spp. [27], *Hyalesthes* spp. [29], *Trissolcus japonicus* [46], and *Hishimonus* spp. [47].

In this study, a specific PCR assay was established to detect the main vector of the SBR disease in sugar beet. The method can be applied to detect *P. leporinus* and discriminate this insect from other morphologically closely related cixiids including *R. quinquecostatus* and *H. obsoletus* [25]. Additionally, the in silico analysis demonstrated that other more distantly related Auchenorrhyncha species, reported from sugar beet fields, will not be detected due to missing target sequence similarity. Supporting the in silico analysis, *P. leporinus* was differentiated from the most common Auchenorrhyncha species reported from German sugar beet fields, including taxonomically distantly related species such as *Empoasca* spp., *F. florii* or *C. placida*.

Immature stages represent the longest time-period of the *P. leporinus* life cycle [8] and morphological description as well as taxonomic keys are missing to precisely discriminate *P. leporinus* immature stages from other cixiids. Molecular methods have been used to identify the immature stages of insects which also expands the monitoring period of insect vectors [29,31]. Similarly, Figure 6 shows that the developed protocol allows detection of all *P. leporinus* immature stages.

We provide a PCR method that reliably (100% detection rate of *P. leporinus* specimens) detects both male and female *P. leporinus*, either from sweep net or sticky trap collection, even if the insects were preserved in 96% ethanol at −20 °C within 24 h after sweep net collection or stored for a short (1–2 weeks) or long time (up to 18 months) on the sticky traps at room temperature before they were preserved in 60 or 70% ethanol. Sticky trap collected specimens were successfully detected without removing sticky trap glue from the insect bodies. Additionally, we established this method with a simple and time saving DNA preparation by grinding specimens in PBS. PBS extracts were successfully used for specific detection of all insect life stages including eggs, nymphs, and adults. Thus, this simple and cheap method is suitable for large scale monitoring assays. Furthermore, sequencing of PCR products is not required due to the species-specificity of this protocol.

The published universal primers (Ron and Calvin) allow the detection of *P. leporinus* only after sequencing the PCR products which is time consuming. In addition, the amplicons for some samples are low in concentration possibly due to the degeneracy of primers. With the lower quality of template DNA in PBS extracts, this degeneracy resulted in weaker signals. Due to the 100% amplification rate of the analyzed *P. leporinus* samples with specific primers, the provided specific primers are more efficient and precise, compared to universal PCR with Ron and Calvin primers. The Ron primer was originally designed for general amplification of lepidopterans, dipterans, coleopterans, thysanopterans, hemipterans, and homopterans [48] and the Calvin primer was originally used to analyze species from the genera Enchenopa and Campylenchia within the family Membracidae [49]. Later, the primer pair Ron and Calvin was used for molecular detection of planthoppers from the infraorder Fulgoromorpha and the families Cixiidae and Delphacidae [44]. Amplification of delphacid DNA with Ron and Calvin primers was also demonstrated in the study of Argüello Caro et al. [37]. In our experiments, cixiid (*P. leporinus*, *R. quinquecostatus*, *H. obsoletus*) and delphacid (*J. pellucida*, *J. obscurella*) DNA was amplified. However, specimens of the families Cicadellidae (exception: *F. florii*) and Membracidae (*S. bisonia*), which belong to the infraorder Cicadomorpha were not detected. Therefore, the Ron and Calvin primer combination was no perfect choice to generally detect all Auchenorrhyncha species by sequencing.

Although UEA3 and UEA8 primers were designed for general *COI* amplification of hemipteran insects [38], due to mismatches, they never amplified *P. leporinus* in this study. This primer pair therefore may only be of use as a negative control for *P. leporinus* detection. Additionally, only one of three *P. alienus* specimens was amplified besides *R. quinquecostatus* and *H. obsoletus* and no other distantly related species, suggesting that this primer pair is not suitable for general *COI* amplification of Auchenorrhyncha species from sugar beet fields.

The evolutionary relationships of numerous cixiid species including *Pentastiridius* sp., *R. quinquecostatus*, and *H. scotti* have been extensively analyzed based on a large fragment (3652 bp in size) of *COI*, Cytochrome b, nuclear 18S rDNA and 28S rDNA genes [43]. Similarly, the *COI* gene (800 bp in size) was used for phylogenetic analysis of cixiids and delphacids including *P. leporinus*, *R. cuspidatus*, and *H. obsoletus* [16,34]. Therefore, the *COI* gene is a suitable gene for differentiation of these species. In our study, we confirmed that a partial *COI* fragment (341 bp) that was specifically amplified from *P. leporinus* in comparison to sequences from *R. quinquecostatus* and *H. obsoletus* can be sufficient to differentiate these morphologically close species.

In addition, phylogenetic analysis for these species based on the generally amplified *COI* fragments (~1000 bp) in comparison to representative species of all Auchenorrhyncha families and subfamilies reported from sugar beet fields confirmed the close morphological features for these three species and that the two close families Cixiidae and Delphacidae can be clearly separated (Figure S4). In several studies, the close relationship between Cixiidae and Delphacidae has been reported [25,43,44] which supports the presented phylogenetic analysis based on the *COI* gene.

In conclusion, we provide here a sensitive, cost- and time-saving molecular method for reliable and specific detection of all immature stages as well as male and female *P. leporinus*, after different methods of planthopper collection and template DNA preparation. This technique has the potential to be used in large scale monitoring assays.

## Figures and Tables

**Table 1:** List of primers used for partial amplification of mitochondrial cytochrome oxidase I gene (*COI*) from *Pentastiridius leporinus*, *Reptalus quinquecostatus* and *Hyalesthes obsoletus*.

Primer Name	Sequence (5′ to 3′)	PCR-Product Size [bp]	Reference
Ron (fw)	GGATCACCTGATATAGCATTCCC	~1000	Argüello Caro [37]
Calvin (rv)	GGRAARAAWGTTAARTTWACTCC		
*P. leporinus* fw1	TTATTGCAGTACCAACAGGT	341	This study
*P. leporinus* rv1	TGTGAAATTTACTCCTGTAAATATAGTAAAG		
UEA3 (fw)	TATAGCATTCCCACGAATAAATAA	~1000	Lunt et al. [38]
UEA8 (rv)	AAAAATGTTGAGGGAAAAATGTTA		

## Data Availability

Data are contained within the article or supplementary material.

## Supplementary Materials

The following supporting information can be downloaded at: https://www.mdpi.com/article/10.3390/insects13110992/s1, Table S1. Table of experimental samples. Table S2. Evolutionary divergence between sequences of selected members of Cixiidae based on the partial *COI* sequence amplified from *P. leporinus* using specific primers in this study and *COI* sequences from the NCBI database of closely related species from the genus *Pentastiridius* and each three species from two taxonomically close genera *Reptalus* and *Hyalesthes.* The sequence obtained in this study is shown in bold. The specifically amplified *COI* fragment shows that intraspecific genetic distance was lower than the interspecific distance. *Catonia carolina* from the Achilidae family and *Tettigometra virescens* from the Tettigometridae family were used as outgroups. Figure S1: Alignment of the specific primers (*P. leporinus* fw1 and *P. leporinus* rv1) to the *COI* of all Auchenorrhyncha species, which were reported from sugar beet fields. Identical nucleotides in the specific primers and the target sequences are marked with dots. Letters highlight nucleotide dissimilarities between primers and target sequences. The number of nucleotide mismatches is summarized in the column at the right side of each alignment. Asterisks mark the positions of conserved nucleotides within primer sequences. Figure S2: Electrophoretic patterns of PCR products show the specific detection of *P. leporinus*. Male adult insects were grinded in PBS for DNA template preparation. The insects were collected from sticky traps and stored on sticky traps for 1–2 weeks before use. In panel A, universal Ron and Calvin primers, in panel B, specific *P. leporinus* primers and in panel C, universal UEA3 and UEA8 primers were used for PCR assay. Lanes 1–3 represent *P. leporinus*; 4–6 *E. pteridis*; 7–9 *E. affinis*; 10–12 *C. placida*; 13–15 *O. ishidae*; 16–18 *R. quinquecostatus*, 19 *H. obsoletus* (after sweep net collection, control); C: negative control (water). The sizes of amplicons are shown on the left side and compared with 1 kb ladder (L). Figure S3: Electrophoretic patterns of PCR products show the specific detection of *P. leporinus*. Male adult insects were grinded in PBS for DNA template preparation. The insects were collected from sticky traps and stored on sticky traps for 1–2 weeks before use. In panel A, universal Ron and Calvin primers, in panel B, specific *P. leporinus* primers and in panel C, universal UEA3 and UEA8 primers were used for PCR assay. Lanes 1–3 represent *P. alienus*; 4–6 *E. decipiens*; 7–9 *F. florii*; 10–12 *J. pellucida*; 13–15 *S. bisonia*; 16–17 *J. obscurella*; 18 *P. leporinus* (control); 19 *R. quinquecostatus* (control); 20 *H. obsoletus* (after sweep net collection, control); C: negative control (water). The sizes of amplicons are shown on the left side and compared with 1 kb ladder (L). Figure S4. Evolutionary relationships of selected members of Cixiidae (*P. leporinus*, *R. quinquecostatus*, and *H. obsoletus*) using the partial sequences of *COI* gene that were PCR amplified with universal primers (Ron and Calvin; UEA3 and UEA8) in this study compared to the available sequences from the NCBI database of representative members of all Auchenorrhyncha families and subfamilies reported from sugar beet fields. The sequences from this study are shown in bold. The percentage of replicate trees in which the associated taxa clustered together in the bootstrap test (1000 replicates) are shown next to the branches. The obtained *COI* fragments were useful to clearly differentiate *P. leporinus*, *R. quinquecostatus*, and *H. obsoletus* from other taxonomically close and far species.
