# Supplementary material for: Molecular Detection of Pentastiridius leporinus, the Main Vector of the Syndrome ‘Basses Richesses’ in Sugar Beet
Source: Insects. 2022 Oct 28;13(11):992. doi: 10.3390/insects13110992 (PMC9695866; doi:10.3390/insects13110992)
Supplement: Supplementary file 1 [file insects-13-00992-s001.zip › insects-1968882-supplementary.pdf]

Supplementary information for the manuscript

Molecular detection of *Pentastiridius leporinus*, the main vector of the syndrome 'basses richesses' in sugar beet

Author information

René Pfitzer[1,2,*], Mark Varrelmann[1], Georgia Hesse[1], Omid Eini[1,*]

[1]Institute of Sugar Beet Research, Holtenser Landstraße 77, 37079 Göttingen, Germany; varrelmann@ifz-goettingen.de (M.V.), hesse@ifz-goettingen.de (G.H.)
Agricultural Entomology, Department of Crop Sciences, Faculty of Agricultural Sciences, University of Göttingen Grisebachstrasse 6, 37077 Göttingen, Germany

*Correspondence: pfitzer@ifz-goettingen.de (R.P.); eini@ifz-goettingen.de (O.E.); Tel.: +49 (0) 551 505 62 24

Tables S1, S2; Figures S1, S2, S3, S4.

Table S1. Table of experimental samples.

Shown in…	No.	Species	Sex/stage	Collection time	Collection site	Collection method	DNA preparation
Fig. 4 (1)	1	*Pentastiridius leporinus*	Adult male	20.06.2020	Untereisesheim	Sweep net	DNeasy kit
Fig. 4 (1)	2	*Pentastiridius leporinus*	Adult male	20.06.2020	Untereisesheim	Sweep net	DNeasy kit
Fig. 4 (1)	3	*Pentastiridius leporinus*	Adult female	20.06.2020	Untereisesheim	Sweep net	DNeasy kit
Fig. 4 (1)	4	*Pentastiridius leporinus*	Adult female	20.06.2020	Untereisesheim	Sweep net	DNeasy kit
Fig. 4 (1)	5	*Reptalus quinquecostatus*	Adult male	20.07.2020	Untereisesheim	Sweep net	DNeasy kit
Fig. 4 (1)	6	*Reptalus quinquecostatus*	Adult male	04.07.2020	Grombach	Sweep net	DNeasy kit
Fig. 4 (1)	7	*Reptalus quinquecostatus*	Adult female	04.07.2020	Untereisesheim	Sweep net	DNeasy kit
Fig. 4 (1)	8	*Reptalus quinquecostatus*	Adult female	04.07.2020	Untereisesheim	Sweep net	DNeasy kit
Fig. 4 (1)	9	*Hyalesthes obsoletus*	Adult male	22.07.2020	Untereisesheim	Sweep net	DNeasy kit
Fig. 4 (1)	10	*Hyalesthes obsoletus*	Adult male	2021	Rheinland Pfalz	Sweep net	DNeasy kit
Fig. 4 (1)	11	*Hyalesthes obsoletus*	Adult female	20.07.2020	Heidelberg	Sweep net	DNeasy kit
Fig. 4 (1)	12	*Hyalesthes obsoletus*	Adult female	22.07.2020	Untereisesheim	Sweep net	DNeasy kit
Fig. 4 (2)	1	*Pentastiridius leporinus*	Adult male	22.06.2020	Untereisesheim	Sticky trap (long term)	DNeasy kit
Fig. 4 (2)	2	*Pentastiridius leporinus*	Adult male	22.06.2020	Untereisesheim	Sticky trap (long term)	DNeasy kit
Fig. 4 (2)	3	*Pentastiridius leporinus*	Adult female	22.06.2020	Bad Rappenau	Sticky trap (long term)	DNeasy kit
Fig. 4 (2)	4	*Pentastiridius leporinus*	Adult female	06.07.2020	Heilbronn	Sticky trap (long term)	DNeasy kit
Fig. 4 (2)	5	*Reptalus quinquecostatus*	Adult male	22.07.2020	Untereisesheim	Sticky trap (long term)	DNeasy kit
Fig. 4 (2)	6	*Reptalus quinquecostatus*	Adult male	22.07.2020	Untereisesheim	Sticky trap (long term)	DNeasy kit
Fig. 4 (2)	7	*Reptalus quinquecostatus*	Adult female	22.07.2020	Untereisesheim	Sticky trap (long term)	DNeasy kit
Fig. 4 (2)	8	*Reptalus quinquecostatus*	Adult female	06.07.2020	Heidelberg	Sticky trap (long term)	DNeasy kit
Fig. 4 (2)	9	*Hyalesthes obsoletus*	Adult male	22.07.2020	Untereisesheim	Sticky trap (long term)	DNeasy kit
Fig. 4 (2)	10	*Hyalesthes obsoletus*	Adult male	22.07.2020	Untereisesheim	Sticky trap (long term)	DNeasy kit
Fig. 4 (2)	11	*Hyalesthes obsoletus*	Adult female	22.07.2020	Untereisesheim	Sticky trap (long term)	DNeasy kit
Fig. 4 (2)	12	*Hyalesthes obsoletus*	Adult female	22.07.2020	Untereisesheim	Sticky trap (long term)	DNeasy kit
Fig. 5 (1)	1	*Pentastiridius leporinus*	Adult male	22.07.2020	Untereisesheim	Sweep net	PBS
Fig. 5 (1)	2	*Pentastiridius leporinus*	Adult male	22.07.2020	Untereisesheim	Sweep net	PBS
Fig. 5 (1)	3	*Pentastiridius leporinus*	Adult female	20.06.2020	Untereisesheim	Sweep net	PBS
Fig. 5 (1)	4	*Pentastiridius leporinus*	Adult female	20.06.2020	Untereisesheim	Sweep net	PBS
Fig. 5 (1)	5	*Reptalus quinquecostatus*	Adult male	20.07.2020	Heidelberg	Sweep net	PBS
Fig. 5 (1)	6	*Reptalus quinquecostatus*	Adult male	22.07.2020	Untereisesheim	Sweep net	PBS
Fig. 5 (1)	7	*Reptalus quinquecostatus*	Adult female	22.07.2020	Untereisesheim	Sweep net	PBS
Fig. 5 (1)	8	*Reptalus quinquecostatus*	Adult female	22.07.2020	Untereisesheim	Sweep net	PBS
Fig. 5 (1)	9	*Hyalesthes obsoletus*	Adult male	04.07.2020	Untereisesheim	Sweep net	PBS
Fig. 5 (1)	10	*Hyalesthes obsoletus*	Adult male	2021	Rheinland Pfalz	Sweep net	PBS
Fig. 5 (1)	11	*Hyalesthes obsoletus*	Adult female	04.07.2020	Untereisesheim	Sweep net	PBS
Fig. 5 (1)	12	*Hyalesthes obsoletus*	Adult female	2021	Rheinland Pfalz	Sweep net	PBS
Fig. 5 (2)	1	*Pentastiridius leporinus*	Adult male	22.06.2020	Untereisesheim	Sticky trap (long term)	PBS
Fig. 5 (2)	2	*Pentastiridius leporinus*	Adult male	22.06.2020	Untereisesheim	Sticky trap (long term)	PBS
Fig. 5 (2)	3	*Pentastiridius leporinus*	Adult female	22.06.2020	Untereisesheim	Sticky trap (long term)	PBS
Fig. 5 (2)	4	*Pentastiridius leporinus*	Adult female	22.06.2020	Dettenheim	Sticky trap (long term)	PBS
Fig. 5 (2)	5	*Reptalus quinquecostatus*	Adult male	06.07.2020	Heidelberg	Sticky trap (long term)	PBS
Fig. 5 (2)	6	*Reptalus quinquecostatus*	Adult male	06.07.2020	Heidelberg	Sticky trap (long term)	PBS
Fig. 5 (2)	7	*Reptalus quinquecostatus*	Adult female	06.07.2020	Heidelberg	Sticky trap (long term)	PBS
Fig. 5 (2)	8	*Reptalus quinquecostatus*	Adult female	06.07.2020	Heidelberg	Sticky trap (long term)	PBS
Fig. 5 (2)	9	*Hyalesthes obsoletus*	Adult male	20.08.2020	Untereisesheim	Sticky trap (long term)	PBS
Fig. 5 (2)	10	*Hyalesthes obsoletus*	Adult male	22.07.2020	Untereisesheim	Sticky trap (long term)	PBS
Fig. 5 (2)	11	*Hyalesthes obsoletus*	Adult female	13.07.2020	Untereisesheim	Sticky trap (long term)	PBS
Fig. 5 (2)	12	*Hyalesthes obsoletus*	Adult female	22.07.2020	Untereisesheim	Sticky trap (long term)	PBS
Fig. 6	1	*Pentastiridius leporinus*	Egg	03.03.2022	Lab. rearing	Collection from rearing	PBS
Fig. 6	2	*Pentastiridius leporinus*	Egg	03.03.2022	Lab. rearing	Collection from rearing	PBS
Fig. 6	3	*Pentastiridius leporinus*	Egg	03.03.2022	Lab. rearing	Collection from rearing	PBS
Fig. 6	4	*Pentastiridius leporinus*	First instar	03.03.2022	Lab. rearing	Collection from rearing	PBS
Fig. 6	5	*Pentastiridius leporinus*	First instar	03.03.2022	Lab. rearing	Collection from rearing	PBS
Fig. 6	6	*Pentastiridius leporinus*	First instar	03.03.2022	Lab. rearing	Collection from rearing	PBS
Fig. 6	7	*Pentastiridius leporinus*	Second instar	03.03.2022	Lab. rearing	Collection from rearing	PBS
Fig. 6	8	*Pentastiridius leporinus*	Second instar	03.03.2022	Lab. rearing	Collection from rearing	PBS
Fig. 6	9	*Pentastiridius leporinus*	Second instar	03.03.2022	Lab. rearing	Collection from rearing	PBS
Fig. 6	10	*Pentastiridius leporinus*	Third instar	03.03.2022	Lab. rearing	Collection from rearing	PBS
Fig. 6	11	*Pentastiridius leporinus*	Third instar	03.03.2022	Lab. rearing	Collection from rearing	PBS
Fig. 6	12	*Pentastiridius leporinus*	Third instar	03.03.2022	Lab. rearing	Collection from rearing	PBS
Fig. 6	13	*Pentastiridius leporinus*	Fourth instar	03.03.2022	Lab. rearing	Collection from rearing	PBS
Fig. 6	14	*Pentastiridius leporinus*	Fourth instar	03.03.2022	Lab. rearing	Collection from rearing	PBS
Fig. 6	15	*Pentastiridius leporinus*	Fourth instar	03.03.2022	Lab. rearing	Collection from rearing	PBS
Fig. 6	16	*Pentastiridius leporinus*	Fifth instar	03.03.2022	Lab. rearing	Collection from rearing	PBS
Fig. 6	17	*Pentastiridius leporinus*	Fifth instar	03.03.2022	Lab. rearing	Collection from rearing	PBS
Fig. 6	18	*Pentastiridius leporinus*	Fifth instar	03.03.2022	Lab. rearing	Collection from rearing	PBS
Fig. 6	19	*Reptalus quinquecostatus*	Adult female	04.07.2020	Untereisesheim	Sweep net	DNeasy kit
Fig. 6	20	*Hyalesthes obsoletus*	Adult female	22.07.2020	Untereisesheim	Sweep net	DNeasy kit

Fig. S2	1	*Empoasca pteridis*	Adult male	16.08.2018	Heidelberg	Sticky trap (short term)	PBS
Fig. S2	2	*Empoasca pteridis*	Adult male	16.08.2018	Heidelberg	Sticky trap (short term)	PBS
Fig. S2	3	*Empoasca pteridis*	Adult male	16.08.2018	Heidelberg	Sticky trap (short term)	PBS
Fig. S2	4	*Empoasca affinis*	Adult male	23.08.2018	Heidelberg	Sticky trap (short term)	PBS
Fig. S2	5	*Empoasca affinis*	Adult male	23.08.2018	Bretten	Sticky trap (short term)	PBS
Fig. S2	6	*Empoasca affinis*	Adult male	23.08.2018	Bad Rappenau	Sticky trap (short term)	PBS
Fig. S2	7	*Cicadula placida*	Adult male	15.06.2018	Heidelberg	Sticky trap (short term)	PBS
Fig. S2	8	*Cicadula placida*	Adult male	15.06.2018	Heidelberg	Sticky trap (short term)	PBS
Fig. S2	9	*Cicadula placida*	Adult male	15.06.2018	Bretten	Sticky trap (short term)	PBS
Fig. S2	10	*Orientus ishidae*	Adult male	10.08.2018	Bretten	Sticky trap (short term)	PBS
Fig. S2	11	*Orientus ishidae*	Adult male	10.08.2018	Bretten	Sticky trap (short term)	PBS
Fig. S2	12	*Orientus ishidae*	Adult male	10.08.2018	Bretten	Sticky trap (short term)	PBS
Fig. S2	13	*Reptalus quinquecostatus*	Adult male	20.07.2018	Bretten	Sticky trap (short term)	PBS
Fig. S2	14	*Reptalus quinquecostatus*	Adult male	27.07.2018	Bretten	Sticky trap (short term)	PBS
Fig. S2	15	*Reptalus quinquecostatus*	Adult male	27.07.2018	Bretten	Sticky trap (short term)	PBS
Fig. S3	1	*Psammotettix alienus*	Adult male	23.08.2018	Heidelberg	Sticky trap (short term)	PBS
Fig. S3	2	*Psammotettix alienus*	Adult male	23.08.2018	Heidelberg	Sticky trap (short term)	PBS
Fig. S3	3	*Psammotettix alienus*	Adult male	23.08.2018	Bretten	Sticky trap (short term)	PBS
Fig. S3	4	*Empoasca decipiens*	Adult male	23.08.2018	Heidelberg	Sticky trap (short term)	PBS
Fig. S3	5	*Empoasca decipiens*	Adult male	23.08.2018	Bad Rappenau	Sticky trap (short term)	PBS
Fig. S3	6	*Empoasca decipiens*	Adult male	23.08.2018	Bad Rappenau	Sticky trap (short term)	PBS
Fig. S3	7	*Fieberiella florii*	Adult male	10.08.2018	Bretten	Sticky trap (short term)	PBS
Fig. S3	8	*Fieberiella florii*	Adult male	10.08.2018	Heidelberg	Sticky trap (short term)	PBS
Fig. S3	9	*Fieberiella florii*	Adult male	10.08.2018	Bretten	Sticky trap (short term)	PBS
Fig. S3	10	*Javesella pellucida*	Adult male	29.06.2022	Bad Rappenau	Sticky trap (short term)	PBS
Fig. S3	11	*Javesella pellucida*	Adult male	29.06.2022	Bad Rappenau	Sticky trap (short term)	PBS
Fig. S3	12	*Javesella pellucida*	Adult male	29.06.2022	Bad Rappenau	Sticky trap (short term)	PBS
Fig. S3	13	*Stictocephala bisonia*	Adult male	23.08.2018	Bretten	Sticky trap (short term)	PBS
Fig. S3	14	*Stictocephala bisonia*	Adult male	23.08.2018	Heidelberg	Sticky trap (short term)	PBS
Fig. S3	15	*Stictocephala bisonia*	Adult male	23.08.2018	Heidelberg	Sticky trap (short term)	PBS
Fig. S3	16	*Javesella obscurella*	Adult male	22.06.2018	Bad Rappenau	Sticky trap (short term)	PBS
Fig. S3	17	*Javesella obscurella*	Adult male	22.06.2018	Bad Rappenau	Sticky trap (short term)	PBS

Table S2. Evolutionary divergence between sequences of selected members of Cixiidae based on the partial *COI* sequence amplified from *P. leporinus* using specific primers in this study and *COI* sequences from NCBI database of closely related species from genus *Pentastiridius* and each three species from two taxonomically close genera *Reptalus* and *Hyalesthes.* The sequence obtained in this study is shown in bold. The specifically amplified *COI* fragment is shows that intraspecific genetic distance was lower than interspecific distance. *Catonia carolina* from the Achilidae family and *Tettigometra virescens* from the Tettigometridae family were used as outgroups.

	**1:**	**2:**	**3:**	**4:**	**5:**	**6:**	**7:**	**8:**	**9:**	**10:**	**11:**	**12:**	**13:**
1: ON094072-*Pentastiridius leporinus*													
2: FN179288-*Pentastiridius leporinus*	0.000												
3: FN179289-*Pentastiridius leporinus*	0.006	0.003											
4: FN179290-*Pentastiridius beieri*	0.051	0.060	0.060										
5: GQ397853-*Reptalus cuspidata*	0.119	0.117	0.113	0.116									
6: GQ397855-*Reptalus melanochaetus*	0.096	0.100	0.094	0.107	0.097								
7: GQ397852-*Reptalus quinquecostatus*	0.104	0.117	0.115	0.121	0.115	0.090							
8: GQ397854-*Reptalus panzeri*	0.108	0.126	0.120	0.125	0.122	0.098	0.091						
9: GU553002-*Hyalesthes obsoletus*	0.134	0.140	0.133	0.135	0.144	0.125	0.137	0.137					
10: GU553003-*Hyalesthes luteipes*	0.162	0.169	0.164	0.157	0.157	0.146	0.146	0.131	0.125				
11: FN428805-*Hyalesthes scotti*	0.166	0.172	0.171	0.159	0.174	0.165	0.173	0.140	0.126	0.064			
12: JQ982584-*Catonia carolina*	0.229	0.247	0.227	0.245	0.257	0.227	0.219	0.242	0.236	0.262	0.245		
13: MK188587-*Tettigometra virescens*	0.741	0.769	0.771	0.766	0.792	0.775	0.756	0.759	0.679	0.735	0.726	0.753	

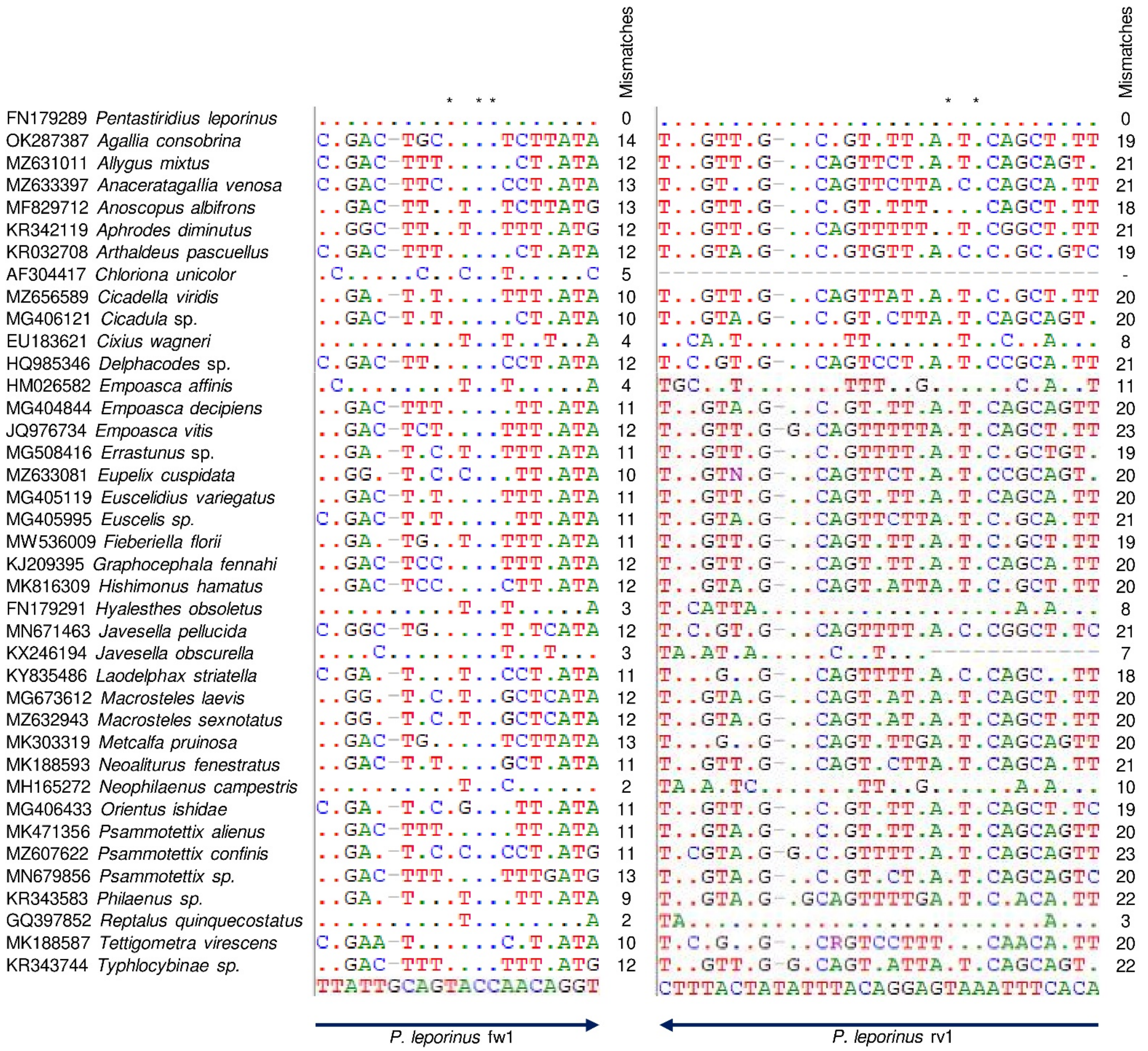

Figure S1. Alignment of the specific primers (*P. leporinus* fw1 and *P. leporinus* rv1) to *COI* of all Auchenorrhyncha species, which were reported from sugar beet fields. Identical nucleotides in the specific primers and the target sequences are marked with dots. Letters highlight nucleotide dissimilarities between primers and target sequences. The number of nucleotide mismatches is summarized in the column at the right side of each alignment. Asterisks mark the positions of conserved nucleotides within primer sequences.

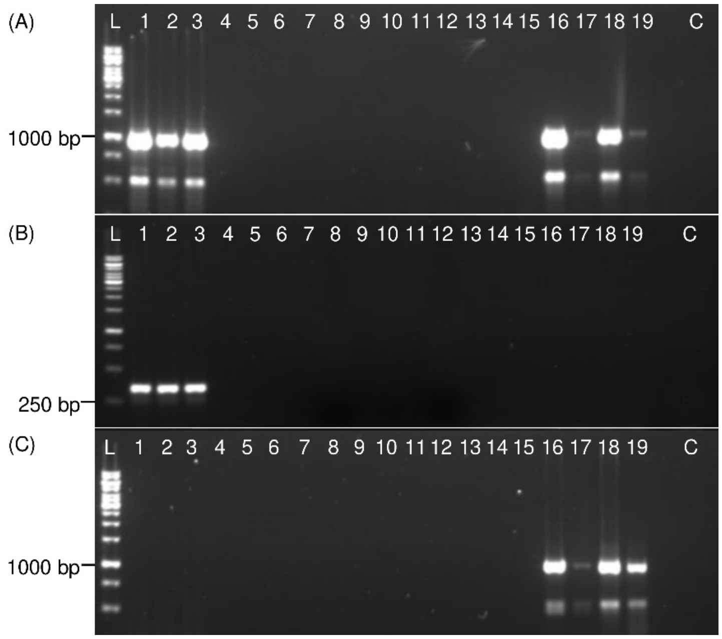

Figure S2. Electrophoretic patterns of PCR products show the specific detection of *P. leporinus*. Male adult insects were grinded in PBS for DNA template preparation. The insects were collected from sticky traps and stored on sticky traps for 1-2 weeks before use. In panel A, universal Ron and Calvin primers, in panel B, specific *P. leporinus* primers and in panel C, universal UEA3 and UEA8 primers were used for PCR assay. Lanes 1-3 represent *P. leporinus*; 4-6 *E. pteridis*; 7-9 *E. affinis*; 10-12 *C. placida*; 13-15 *O. ishidae*; 16-18 *R. quinquecostatus*, 19 *H. obsoletus* (after sweep net collection, control); C: Negative control (water). The sizes of amplicons are shown on the left side and compared with 1 kb ladder (L).

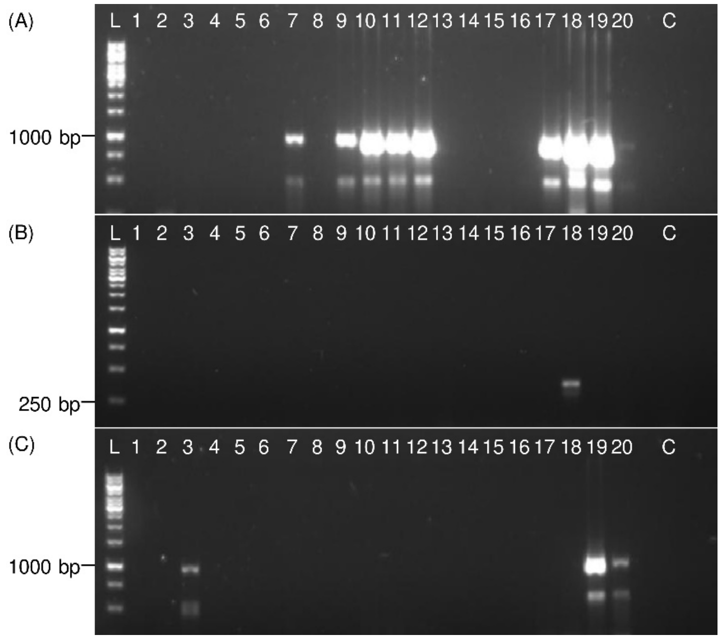

Figure S3. Electrophoretic patterns of PCR products show the specific detection of *P. leporinus*. Male adult insects were grinded in PBS for DNA template preparation. The insects were collected from sticky traps and stored on sticky traps for 1-2 weeks before use. In panel A, universal Ron and Calvin primers, in panel B, specific *P. leporinus* primers and in panel C, universal UEA3 and UEA8 primers were used for PCR assay. Lanes 1-3 represent *P. alienus*; 4-6 *E. decipiens*; 7-9 *F. florii*; 10-12 *J. pellucida*; 13-15 *S. bisonia*; 16-17 *J. obscurella*; 18 *P. leporinus* (control); 19 *R. quinquecostatus* (control); 20 *H. obsoletus* (after sweep net collection, control); C: Negative control (water). The sizes of amplicons are shown on the left side and compared with 1 kb ladder (L).

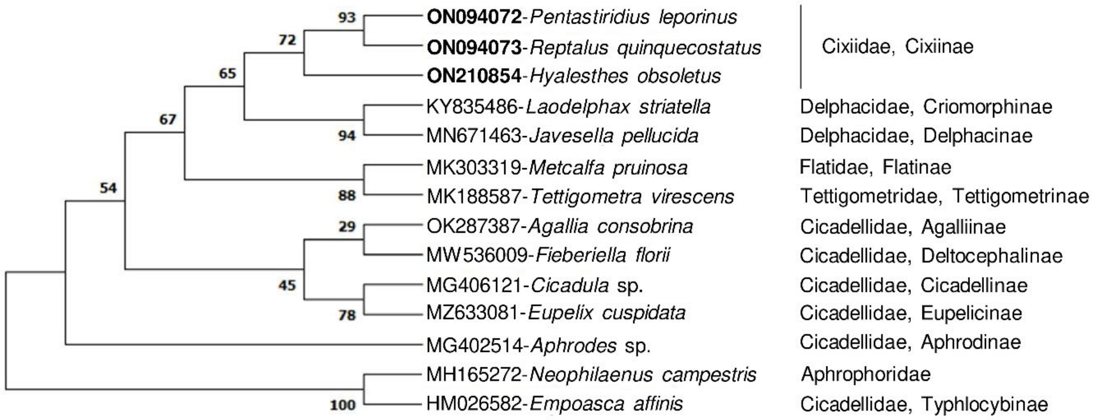

Figure S4. Evolutionary relationships of selected members of Cixiidae (*P. leporinus*, *R. quinquecostatus*, and *H. obsoletus*) using the sequences of *COI* gene that were PCR amplified with universal primers (Ron and Calvin; UEA3 and UEA8) in this study compared to the available sequences from NCBI database of representative members of all Auchenorrhyncha families and subfamilies reported from sugar beet fields. The sequences from this study are shown in bold. The percentage of replicate trees in which the associated taxa clustered together in the bootstrap test (1000 replicates) are shown next to the branches. The obtained *COI* fragments were useful to clearly differentiate *P. leporinus*, *R. quinquecostatus*, and *H. obsoletus* from other taxonomically close and far species.
